# A Microglial Signature Directing Human Aging and Neurodegeneration-Related Gene Networks

**DOI:** 10.3389/fnins.2019.00002

**Published:** 2019-01-24

**Authors:** Shradha Mukherjee, Christine Klaus, Mihaela Pricop-Jeckstadt, Jeremy A. Miller, Felix L. Struebing

**Affiliations:** ^1^Health Informatics Advanced Science Masters Program, Arizona State University, Tempe, AZ, United States; ^2^Department of Neurology, University of California, Los Angeles, Los Angeles, CA, United States; ^3^Department of Bioinformatics, University of California, Los Angeles, Los Angeles, CA, United States; ^4^Neural Regeneration Group, Institute of Reconstructive Neurobiology, University of Bonn, Bonn, Germany; ^5^Institute for Medical Informatics and Biometry, Faculty of Medicine “Carl Gustav Carus”, TU Dresden, Dresden, Germany; ^6^Allen Institute for Brain Science, Seattle, WA, United States; ^7^Department of Translational Brain Research, German Center for Neurodegenerative Diseases, Munich, Germany; ^8^Center for Neuropathology and Prion Research, Ludwig Maximilian University of Munich, Munich, Germany; ^9^Department of Ophthalmology, Emory University, Atlanta, GA, United States

**Keywords:** aging, neurodegeneration, microglia, Alzheimer, Parkinson, bioinformatics, gene networks, WGCNA

## Abstract

Aging is regarded as a major risk factor for neurodegenerative diseases. Thus, a better understanding of the similarities between the aging process and neurodegenerative diseases at the cellular and molecular level may reveal better understanding of this detrimental relationship. In the present study, we mined publicly available gene expression datasets from healthy individuals and patients affected by neurodegenerative diseases (Alzheimer’s disease, Parkinson’s disease, and Huntington’s disease) across a broad age spectrum and compared those with mouse aging and mouse cell-type specific gene expression profiles. We performed weighted gene co-expression network analysis (WGCNA) and found a gene network strongly related with both aging and neurodegenerative diseases. This network was significantly enriched with a microglial signature as imputed from cell type-specific sequencing data. Since mouse models are extensively used for the study of human diseases, we further compared these human gene regulatory networks with age-specific mouse brain transcriptomes. We discovered significantly preserved networks with both human aging and human disease and identified 17 shared genes in the top-ranked immune/microglia module, among which we found five human hub genes *TYROBP, FCER1G, ITGB2, MYO1F, PTPRC*, and two mouse hub genes *Trem2* and *C1qa*. Taken together, these results support the hypothesis that microglia are key players involved in human aging and neurodegenerative diseases, and suggest that mouse models should be appropriate for studying these microglial changes in human.

## Introduction

Healthy aging is turning into a major societal challenge. Worldwide, over 960 million people are 60 years or older and this number is expected to double by the year 2050 (United Nations, Department of Economic and Social Affairs, Population Division, 2017). Furthermore, the risk of developing neurodegenerative disease increases with age (Niccoli and Partridge, 2012). The United States medical health system is already spending over $800 billion dollars for the therapy of disorders related to aging (Gooch et al., 2017), of which approximately one third is used for dementia-related diseases. While great advances have been made in unraveling the underlying molecular processes of the aging brain (Kirkwood, 2011; Mattson and Arumugam, 2018), so far only symptomatic treatments have been established, while curative treatments are still lacking.

Some neurodegenerative disorders are clearly of a heritable nature, but unfortunately, most of them are more complex and caused in a polygenic or even omnigenic manner (Boyle et al., 2017). Although genome-wide association studies (GWAS) have improved our understanding of common risk genes for neurodegenerative diseases (Bertram and Tanzi, 2009; Ramanan and Saykin, 2013; Pihlstrøm et al., 2017; Pottier et al., 2018), a clearer understanding of how ‘healthy aging’ turns into neurological impairments (‘poor aging’) is needed. The expression of certain disease-associated genes is known to change with time and there might be a critical transition state before the onset of a neurodegenerative disease (Fjell et al., 2014), however, it is not clear whether complete networks break down or whether certain cell types drive healthy aging into poor aging (Kumar et al., 2013; Carmona-Gutierrez et al., 2016; Plaza-Zabala et al., 2017).

In the recent years, time-effective and low-cost high-throughput sequencing made it possible to analyze whole transcriptomes of humans and rodents even on a single-cell level. Here, we use a systems biology approach to compare publicly available gene expression datasets from healthy humans and patients affected by complex neurodegenerative disorders [Alzheimer’s disease (AD), Huntington’s disease (HD), and Parkinson’s disease (PD)] across a broad spectrum of ages to address the question how ‘healthy aging’ turns into ‘poor aging.’ We obtain relevant transcriptional modules based on their co-expression relationship and further demonstrate that some modules are enriched in specific cell types such as microglia.

Since studying the human brain transcriptome is limited to post-mortem tissue analyses, most knowledge about brain aging comes from mouse or lower vertebrate models (Rosenthal and Brown, 2007). However, the cognitive decline in these models, which is the most accurate clinical hallmark for aging and neurodegeneration, is less severe than what is seen in humans. Thus, we further contrast our findings of human aging to data from healthy aging mouse brains and found similarly preserved networks and enrichments in cell type signature genes. Our study highlights microglia signatures in the center of common biological processes in human aging and neurodegenerative disease, as well as mouse aging.

## Materials and Methods

### Data Availability

Our computational pipeline used in this publication is open-sourced and available at: https://github.com/smukher2/GithubFrontiersNeurosciDec2018.

### Criteria for Selection and Data Sources

Gene expression profile (microarray and RNA-seq) datasets available on Gene Expression Omnibus (GEO) were selected. For analysis of aging and disease, datasets from studies with multiple time points across age for control and neurodegenerative disease brains were selected. Human AD, HD, and PD disease studies that met these selection criteria, GSE33000 [microarray, prefrontal cortex, brain (Narayanan et al., 2014)] and GSE43490 [microarray, substantia nigra, brain (Corradini et al., 2014)] were analyzed. For cross species comparison, mouse hippocampus RNA-seq datasets from studies with multiple time points across age were selected: GSE61915 (Stilling et al., 2014), GSE73503 (Aaronson and Rosinski, 2015) and GSE83931 (Bundy et al., 2017). To investigate cell type signatures associated with aging and neurodegenerative diseases, an RNA-seq dataset taken from mouse brain (GSE52564, Zhang et al., 2014) encompassing microglia, astrocyte, neuron, oligodendrocyte precursors, newly formed oligodendrocytes, mature oligodendrocytes and endothelial cells was utilized.

### Microarray Data Preparation and Annotation

Two human aging and neurodegenerative disease microarray datasets were downloaded from GEO using GEOquery (Davis and Meltzer, 2007): GEO33000 (dual-color) and GEO43490 (single-color). Gene expression matrices were obtained from GEO, which consisted of normalized log_10_ (Cy5/Cy3) test/reference for GSE33000 (raw ‘CEL’ files not available) and normalized log_2_ signal intensity for GSE43490. Gene expression matrices were converted to linear space, quantile normalized with Lumi 2.32.0 (Du et al., 2008), and then log_2_+1-scaled. ProbeIDs were annotated using the appropriate GPL file downloaded from GEO. The output metadata and gene expression files were merged in the same order. This resulted in a combined sample size of 637 human aging and human neurodegenerative disease data points.

### RNA-Seq Data Preparation and Annotation

Raw fastq RNA-seq reads were obtained for mouse hippocampus aging and mouse cerebral cortex cell types. GSE83931 and GSE73503 were total RNA samples prepared for single-end (NEBnext Ultra mRNA library preparation kit) and paired-end sequencing (standard Illumina library preparation protocols), respectively. GSE61915 and GSE52564 were polyA samples prepared for single-end sequencing using the TruSeq library preparation kit. Fastq files were processed on Cyverse’s Atmosphere cloud-computing platform led by Arizona State University, Tempe, AZ, United States (Goff et al., 2011; Merchant et al., 2016). Briefly, raw reads were obtained with sratoolkit.2.9.1 (Leinonen et al., 2011) and quality was evaluated with fastqc_v0.11.7 (Brown et al., 2017). We employed the tophat 2.1.1 aligner (Trapnell et al., 2009) to map reads to the mouse reference genome mm10 (GRCm38.92) from Ensembl (Zerbino et al., 2018) with the annotation file from GENCODE (GRCm38.92), using recommended/default tophat parameters for paired-end and single-end reads (parameters: –b2 –very-fast –no-coverage-search –no-novel-juncs), which generated bam files from samples aligned at ∼90% or more. The mapped bam files were sorted with samtools-0.1.19 (Li et al., 2009). Sorted bam files were processed with HTSeq 0.10.0 (Anders et al., 2015) to obtain counts of reads aligned to genes (parameters: -r pos –t gene_name). The output metadata and gene expression htseq-count files were merged, normalized to the log_2_+1 scale using edgeR 3.22.5 (Robinson et al., 2009) and filtered for a cpm library size of >1 count in ≥3 samples, which resulted in a combined mouse aging sample size of 71. Additionally, for GSE52564, the sorted bam files were processed with cuffdiff2 from cufflinks_2.1.1-4 (Trapnell et al., 2012) to obtain a list of differentially expressed genes or cell type signature genes as defined by a fold change >20 in comparison of the given cell type to all other cell types. Our list was comparable to the previously published cell type signature gene list obtained from this same GSE52564 dataset (Zhang et al., 2014).

### Adjusting for Surrogate Variables Including Batch Effects

We used the Surrogate Variable analysis (SVA 3.28.0) R package (Leek and Storey, 2007; Leek et al., 2012) for removal of confounding effects from the combined human microarray expression data and combined mouse RNA-seq expression data (SVA model: ∼study or batch + gender + disease + age; null model: ∼study or batch + gender). Because tissue and study/batch were highly correlated, we only used one of these confounding factors (study/batch) for SVA modeling to avoid multicollinearity. For the estimation of variable effects by SVA the “be” method was used. SVA itself does not give the adjusted or normalized expression values and is therefore used in combination with the linear regression model (LM) fitting function from the Limma 3.36.5 R package (Ritchie et al., 2015). This resulted in SVA+LM normalized expression for age and neurodegenerative disease effects in human and mouse merged data, which was then used as input for weighted gene co-expression network analysis (WGCNA) separately for mouse and human.

### Data Used for WGCNA

Matching of unique microarray gene symbols between the human SVA+LM normalized datasets (GSE33000 and GSE43490) left 7301 common genes that were run through the WGCNA meta-analysis pipeline. For the mouse data, gene symbols were first mapped to human gene symbols using biomaRt [version 2.36.1, (Kasprzyk, 2011)], then arranged by expression variability in descending order, and finally the same number of genes as in human (7301) were used as input for WGCNA.

### Meta-Analysis of Gene Expression Networks Using WGCNA

We conducted WGCNA meta-analysis (Miller et al., 2010) and utilized other functions of the WGCNA (Langfelder et al., 2008) R package (version 1.66) to compare co-expressed gene networks between (1) human aging and human neurodegenerative disease and (2) mouse aging. For both, genes were hierarchically clustered and groups of co-expressed genes (modules) were generated using the dynamic tree cutting algorithm with the following major parameters: soft power = 12, TOMType = “signed,” deepSplit = 2, pamStage = TRUE, cutHeight = 0.99 and minClusterSize = 30-3^∗^deepSplit. Each module was assigned a unique color label. Hub genes were identified for each module using gene-module association K_ME_ values. Statistical significance was determined by regressing the traits onto the eigengene of the module, also called trait analysis (Plaisier et al., 2009), which revealed modules that were significantly associated with age (for mouse and human datasets) and disease (for human datasets).

### Identification of Cell Type Signatures in Gene Modules

To identify cell type signatures enriched in the aging and neurodegenerative disease preserved modules in mouse and human, we utilized the inbuilt hypergeometric test function “userListEnrichment” from the WGCNA 1.66 R package. We found overlapping genes between WGCNA modules and the set of cell type signature genes by pairwise comparison, and determined the significance of the overlaps with *p*-values adjusted with Bonferroni’s method for multiple testing. Cell type signatures of microglial hub genes were confirmed using the RNA-Seq Data Navigator tool, which is freely available online as part of the Allen Cell Types Database (© 2015 Allen Institute for Brain Science)^1^. This was done by inputting hub genes in the “Gene Selection” box and building a “Group Plot” using default parameters. Cluster names were modified from the online tool to highlight broad cell types of origin, but no modifications were made to the resulting gene expression signatures. For mouse, cell types were grouped by “subclass” for ease of visualization.

### Cross-Species Comparison of Networks

We then assessed which modules, if any, were preserved between the human and mouse networks using module preservation Z statistics and *p*-values as described in the WGCNA package documentation (Langfelder et al., 2011). Similarly, we used this hypergeometric test described in the previous section to identify modules in the human and mouse networks with a significant number of overlapping genes. Together, these strategies allowed us to identify mouse and human modules that represent equivalent cell types of biological processes.

### KEGG Pathway and Reactome Pathway Analysis of Gene Expression Networks

The modules that were most preserved were annotated and characterized with KEGG pathways and Reactome pathways with the R package enrichR 1.0 (Jawaid, 2017). The top hits of the package output ‘combined scores’ and adjusted *p*-values were used to determine significance.

## Results

**FIGURE 1:**
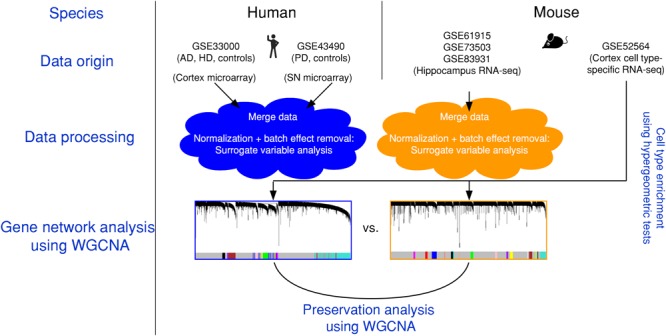
Experimental design and data pre-processing. Two microarray-based gene expression datasets of human patients including three neurodegenerative diseases and age-matched controls were combined and analyzed. Simultaneously, three RNA-sequencing-based gene expression datasets from mouse hippocampus were combined. The human and the mouse datasets were corrected for batch effects and gene networks were constructed with WGCNA. Species-specific networks were checked for overlap with cell type-specific RNA-sequencing data. AD, Alzheimer’s disease; HD, Huntington’s disease; PD, Parkinson’s disease; SN, substantia nigra.

The present article comprises results from the Systems Genetics of Neurodegeneration Summer School held in 2017 and combines analyses from multiple published high-throughput transcriptome datasets (Table 1) that were re-analyzed according to the experimental diagram shown in Figure 1.

**Table 1:** Overview of the datasets used.

Dataset	Tissue	Design	Platform	GEO accession	Reference ID #	Reference
DLPFC (BA9) brain tissues of AD patients, HD patients, and non-demented controls samples	Postmortem prefrontal cortex from Harvard Brain tissue resource center	624 individual DLPFC samples were profiled against a common DLPFC pool constructed from the same set of samples	Agilent 44K array (GPL4372)	GSE33000	GSM1423780 to GSM1424403	Narayanan et al., 2014
Transcriptomic profiles of controls and Parkinson’s disease patients	Postmortem samples of dorsal motor vagal nucleus, locus coeruleus, and substantia nigra from controls and PD subjects from Brain Bank of the Brazilian Aging Brain Study Group, BEHEEC-FMUSP Note: In our present study, we only used the substantia nigra tissue data	Transcriptomic profiles of controls and Parkinson’s disease patients were compared using SAM test for LC or Wilcoxon Mann–Whitney test for SN and VA (*p* < 0.005 and *p* < 0.01, respectively) in order to identify differentially expressed transcripts	Agilent-014850 Whole Human Genome Microarray 4x44K G4112F (Probe Name version)	GSE43490	GSM1294118 to GSM1294130	Corradini et al., 2014
Transcriptome database of eight cell types of mouse cerebral cortex	Isolated and purified neurons, astrocytes, oligodendrocyte precursor cells, newly formed oligodendrocytes, myelinating oligodendrocytes, microglia, endothelial cells, and pericytes from mouse cerebral cortex (different mouse lines)	RNA isolated from purified cell samples using a highly sensitive algorithm to detect alternative splicing in each gene were used to identify cell type enriched genes	Illumina HiSeq 2000 (*Mus musculus*) (GPL13112)	GSE52564	GSM1269903 to GSM1269916	Zhang et al., 2014
Transcriptome data of young and old mouse hippocampus	Mouse hippocampus of 3, 24, and 29 months old C57BL/6J mice	polyA-enriched RNA extracted from mouse hippocampus in three different age groups [3M vs. 24M (*n* = 5–6, single-end sequencing) and 3M vs. 29M (*n* = 3, paired-end sequencing)]	Illumina HiSeq 2000 (*Mus musculus*) (GPL13112)	GSE61915	SRR1593496 to SRR1593512	Stilling et al., 2014
Transcriptome data of knock-in mouse models of Huntington’s disease	Mouse hippocampus of 2, 6, and 10 months old knock-in mice with CAG lengths of 20, 80, 92, 111, 140, 175 along with littermate control wild-type animals Note: In our present study, we only used WT data	mRNA expression profile of male and female (*n* = 4 each) mice of three different age points and seven different conditions (CAG lengths = normal, 20, 80, 92, 111, 140, 175)	Illumina HiSeq 2000 (*Mus musculus*) (GPL13112)	GSE73503	SRR2531532 to SRR2531555	Aaronson and Rosinski, 2015
Transcriptome data of the developing mouse hippocampus	Mouse hippocampus of 1, 2, and 4 months old B6 mice	Hippocampal mRNA from 1, 2, and 4 months old male and female B6 mice were analyzed by RNA sequencing of five biological replicates	Illumina HiSeq 2500 (*Mus musculus*) (GPL17021)	GSE83931	SRR3734796 to SRR3734825	Bundy et al., 2017

**FIGURE 2:**
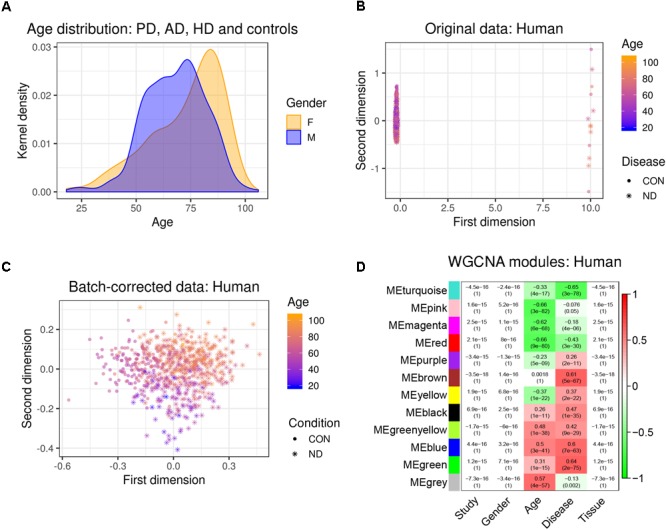
Removal of batch effects and gene network construction in human aging and neurodegeneration. **(A)** Age and gender distribution for the combined human dataset. **(B,C)** The different microarray datasets showed a strong batch effect, which was corrected using SVA to remove confounders while retaining age and disease as main discriminators (CON, healthy controls; ND, neurodegeneration). **(D)** WGCNA modules: Each row corresponds to one gene co-expression network (labeled by color). Numbers in the table indicate the Pearson correlation coefficient *r* and the associated *p*-values in parentheses. Coloring of the table encodes the correlation between each phenotype and each module eigengene (scale on the right).

We first combined two large microarray expression datasets for three neurodegenerative disorders: AD and HD with their respective controls (Narayanan et al., 2014), and PD with its respective controls (Corradini et al., 2014) (Figure 1). These gene expression data were either taken from human post-mortem prefrontal cortex (AD + HD), or human post-mortem substantia nigra (PD). We obtained one large dataset of *n* = 637 patients and controls with balanced gender distribution and an age range of 18–106 years (Figure 2A). Since these data were originally gathered in different laboratories and originated from different brain areas, there was a strong batch effect (Figure 2B). Furthermore, neurodegenerative disease is known to be a strong confounder of aging, and vice-versa (Hung et al., 2010). Thus, we leveraged Surrogate Variable Analysis (Leek et al., 2012) and linear regression models to remove confounders (batch/tissue and gender, see the section “Materials and Methods,” Figure 1). Dimensionality reduction techniques showed that age and neurodegeneration were still retained as a primary components for further analysis, while there were no visible batch effects (Figure 2C). To define and contrast gene networks that were conserved between human aging and neurodegenerative disease, a co-expression gene network was then constructed that consisted of a normalized and combined AD, HD, and PD samples coded as “neurodegeneration” and respective controls. This approach increased power, but had the disadvantage to mask any potential tissue-specific networks.

Hierarchical clustering of expression data using WGCNA partitioned this large human data set into 12 modules (Figure 2D). These modules, which are designated in different colors, can be thought of as functionally different compartments of the human brain transcriptome, forming groups of highly interconnected transcripts that may shape a pathway that is relevant to aging and/or neurodegenerative disease (Fuller et al., 2007; Langfelder et al., 2008; Struebing et al., 2016, 2018). No module had any relationship to the confounding variables study, gender or tissue, validating our batch adjustment strategy (Figure 2D). In order to gain more insight on the biological function of these modules, we overlapped genes within each of the previously found 12 co-expression modules with a signature gene list that was specific for several cortical cell types. These included neurons, astrocytes, oligodendrocyte precursor cells, newly formed oligodendrocytes, myelinating oligodendrocytes, microglia, endothelial cells and pericytes (Zhang et al., 2014). We then tested each module-cell type association for significance using a hypergeometric test. From all mutual comparisons, only three reached significance after adjusting for multiple comparisons (Supplementary Table S1): The human “black” module was related to microglia (*p* = 8.00e-60), whereas the human “blue” module was enriched in endothelial cells (*p* = 1.10e-7), and the human “turquoise” module was related to neurons (*p* = 3.85e-05). Notably, the “black” microglia module had a significant positive relationship to human aging (*r*^2^ = 0.26, *p* = 1e-11), which only became stronger in neurodegeneration (*r*^2^ = 0.47, *p* = 1e-35; Figure 2D). Thus, our analysis revealed that gene networks related to aging and neurodegeneration showed the strongest functional association with microglia, the resident immune cells of the brain. Furthermore, the human “blue” endothelial module was positively related to age (*r*^2^ = 0.5, *p* = 3e-41), whereas the human “turquoise” neuron module was negatively correlated with age (*r*^2^ = -0.33, *p* = 4e-17) (Figure 2D). Both of these associations also became stronger in neurodegeneration (human “blue” *r*^2^ = 0.6, *p* = 7e-63; human “turquoise” *r*^2^ = -0.65, *p* = 3e-78), suggesting that the transcriptome changes for aging and neurodegeneration are similar, albeit exaggerated in the latter.

To further test how human aging could be compared to aging in mice, we again constructed a gene co-expression network using a merged data set from three public RNA-seq data sets taken from healthy mouse hippocampi, covering an age range from 1 month to 29 months of age (1, 2, 3, 6, 10, 24, and 29 months, total *n* = 71). We ensured to only include data that was taken from the same mouse strain (C57BL/6J).

**FIGURE 3:**
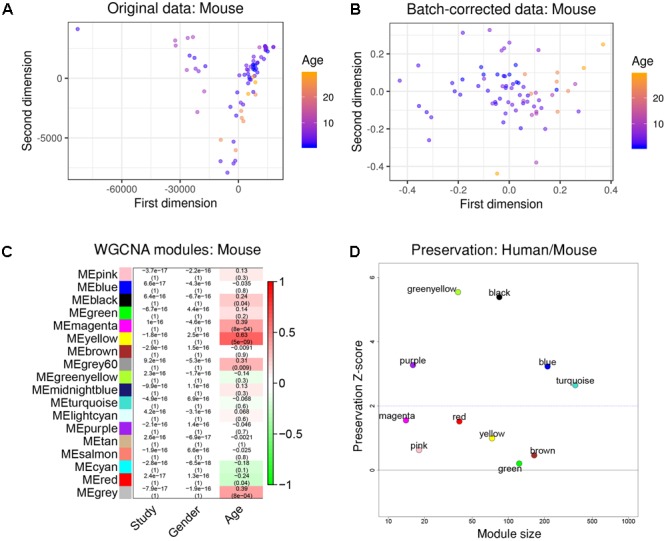
Removal of batch effects and gene network construction in mouse aging. **(A,B)** Dimensionality reduction of the three combined mouse RNA-seq datasets suggested a strong batch effect that was successfully mitigated by the chosen batch correction method. **(C)** WGCNA modules: Each row corresponds to one gene co-expression network (labeled by color). Numbers in the table indicate the Pearson correlation coefficient *r* and the associated *p*-values in parentheses. Coloring of the table encodes the correlation to the phenotype. The mouse “yellow” module was highly related to aging and also showed a significant enrichment in microglia. **(D)** Preservation analysis of mouse and human gene networks using human colors derived from Figure 2D. The human “black” module shows the strongest preservation in mice.

After re-mapping of raw reads (Langfelder et al., 2011), batch effects were successfully removed using the same strategy as previously demonstrated (Figures 3A,B). WGCNA partitioned the combined dataset into 18 modules, including some that depended on mouse age (Figure 3C). Just as with the human data, we also performed cell type-specific enrichment for the mouse aging modules. Among a few significant enrichments, we again found a strong overrepresentation of microglial genes, this time in the mouse “yellow” module (*p* = 2.28e-37; Supplementary Table S2). This module also had the strongest positive correlation to mouse age (*r*^2^ = 0.63, *p* = 5e-9; Figure 3C). Other associations included oligodendrocyte- or neuron- enriched modules, however, their relations were not as prominent as the microglia module (Supplementary Table S2). These results indicate that age-related transcriptome changes involve microglia in humans and mice alike.

**FIGURE 4:**
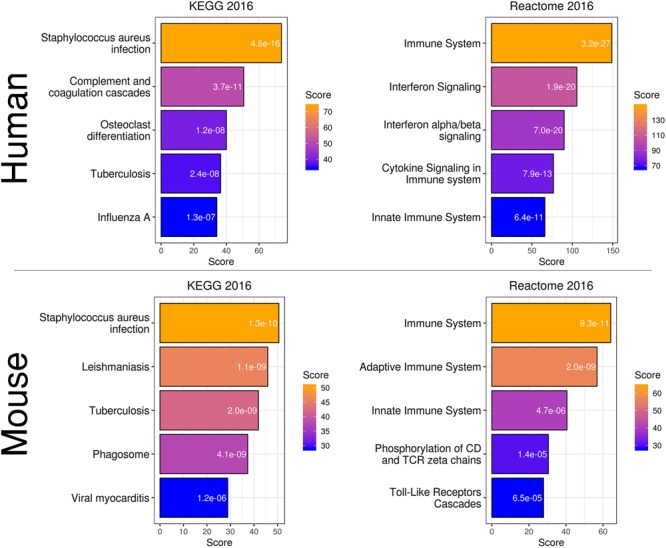
Gene expression classification and pathway analysis of human “black” and mouse “yellow” microglia-specific modules. Both species showed a similar enrichment in pathways related to infection and the immune system.

To more directly compare these networks and potentially identify common transcriptome drivers, we performed module preservation analysis (Langfelder et al., 2011), which can assess the degree to which the co-expression structure of genes in human modules was conserved in mouse (Figure 3D). Previous microarray studies have shown such an association between human and chimpanzee brains (Oldham et al., 2006). The preservation between species was determined by calculating the Z-score, where a score between 5 and 10 is considered to be a moderate, higher-than-expected preservation of the module (Miller et al., 2010). Interestingly, we found the most significant module preservation in the human “black” module, which was enriched in microglial genes (*p* = 8e-60). This module also demonstrated a highly significant hypergeometric enrichment (*p* = 8e-16; Supplementary Table S3) with the mouse “yellow” module, which we previously showed was enriched with microglia genes as well (*p* = 2.28e-37). Functional annotations of the microglia-related human “black” and mouse “yellow” modules showed similar enrichments in immune system-related processes (Figure 4). Besides the Reactome identifiers “immune system” (human *p* = 3.2e-27, mouse *p* = 9.33e-11) and “innate immune system” (human *p* = 6.4e-11, mouse *p* = 4.7e-06), several infectious disease pathways from the Kyoto Encyclopedia of Genes and Genomes (KEGG) were identified. These results suggest that the main actors driving the transcriptome changes associated with aging in human and mice are immune system-related genes expressed in microglia.

**FIGURE 5:**
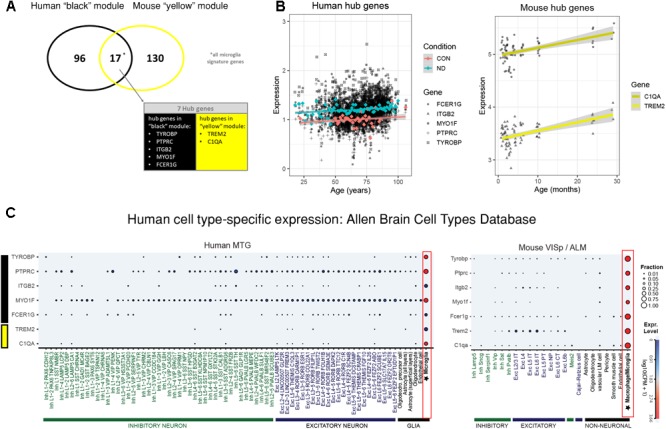
Microglia-specific hub genes in mouse and human and enrichment with the Allen Brain Cell Types Database. **(A)** The human “black” module and the mouse “yellow” module shared 17 genes, out of which five were hub genes in human and two were hub genes in mouse. All 17 shared genes were microglia signature genes as confirmed with the published cell type signature genes. **(B)** These hub genes correlated positively with age in both mouse and human, while mean expression is higher in human neurodegeneration (ND) than in controls (CON). Each gray dot represents one sample and gene, whereas the blue (ND) and the red (CON) dots indicate means and the colored lines represent a linear fit with a gray-shaded 95% confidence interval. **(C)** The hub genes were enriched in human (*left*) and mouse (*right*) microglia. Gene symbols are given on the *y*-axis, while different cell types as defined in the Allen Cell Types Database are displayed on the *x*-axis. Dot size and color correspond to the fraction of cells in a cluster expressing a given gene and the median gene expression, respectively. CPM, counts per million; MTG, middle temporal gyrus; VISp, primary visual cortex; ALM, anterior lateral motor cortex; Inh, inhibitory neurons; Exc, excitatory neurons; oligodendro, oligodendrocytes, LM, leptomeningeal; IT, intratelencephalic; PT, pyramidal tract; NP, near-projecting; CT, corticothalamic. Microglia are highlighted with a red box.

The human “black” module and the mouse “yellow” module had 17 genes in common (Figure 5A and Supplementary Table S3). All of these overlapping genes were also microglia signature genes, out of which seven were among the top ten hub genes in human or mouse (Tables 2, 3 and detailed: Supplementary Tables S3, S4), meaning that they displayed some of the most representative gene expression patterns of the module as a whole and are likely good markers of microglia or their associated processes. *FCER1G* (transmembrane signaling receptor), *ITGB2* (=*CD18*, subunit of complement receptor 3), *MYO1F* (required in cytoskeleton remodeling and migration) (Kalhammer and Bähler, 2000; Chen and Iijima, 2012; Maravillas-Montero and Santos-Argumedo, 2012), *PTPRC* (=*CD45* or *B220*, signaling molecule) and *TYROBP* (=*DAP12*, activatory adaptor protein) were hub genes in the human “black” module, while *C1qa* (subunit of complement component *C1q*) and *Trem2* (transmembrane signaling receptor) were hub genes within the mouse “yellow” module (Figure 5A). By directly interrogating their expression changes during the aging process, we found significant associations of these hub genes with human aging in the “black” module (Figure 5B and Tables 2, 3). All of these genes were strongly enriched in microglia, as evidenced by comparison with our cell type-specific gene list (Supplementary Tables S1, S2). Cross-referencing them with RNA-seq data from the Allen Cell Types Database (Figure 5C) further confirmed the cellular identity of these modules. These results suggest that even though gene networks enriched in microglial signatures that potentially direct human and mouse aging show significant overlap and are preserved, they are controlled by different regulatory hubs in the two species.

**Table 2:** Overview of top 10 hub genes in human “black” module.

Rank	Gene	CorrDisease	PvalDisease	CorrAge	PvalAge	CorrGene	PvalGene
1	TBXAS1	0.399	1.03E-25	0.200	3.56E-07	0.882	6.87E-210
2	**TYROBP**	0.397	2.03E-25	0.085	0.032	0.872	5.07E-199
3	**PTPRC**	0.327	2.35E-17	0.244	4.41E-10	0.871	3.43E-198
4	**ITGB2**	0.395	3.01E-25	0.194	8.27E-07	0.859	1.76E-186
5	**MYO1F**	0.380	2.76E-23	0.102	0.010	0.846	9.05E-176
6	LST1	0.286	1.94E-13	0.157	7.10E-05	0.841	2.36E-171
7	CYBA	0.435	9.96E-31	0.282	4.43E-13	0.840	1.59E-170
8	SLC7A7	0.371	3.69E-22	0.133	0.001	0.837	4.71E-168
9	RNASET2	0.403	2.65E-26	0.189	1.63E-06	0.835	3.72E-167
10	**FCER1G**	0.456	5.16E-34	0.246	2.95E-10	0.829	2.60E-162

*Rank, rank of hub gene (ordered descending by CorrGene); gene, gene symbol; CorrDisease, Pearson correlation of human “black” module eigengene with a numeric vector indicating whether the sample comes from a control (0) or disease (1) donor; PvalDisease, P-value of correlation in CorrDisease; CorrAge, Pearson correlation of human “black” module eigengene with donor age; PvalAve, P-values of correlation in CorrAge; CorrGene, Pearson correlation of human “black” module eigengene with expression level of the gene in the “Gene” column; PvalGene, P-values of correlation in CorrGene. Genes labeled in bold are microglia signature genes that are shared by the human “black” module and the mouse “yellow” module.*

**Table 3:** Overview of top 10 hub genes in mouse “yellow” module.

Rank	Gene	CorrAge	PvalAge	CorrGene	PvalGene
1	C4B	0.652	7.39E-10	0.940	6.58E-34
2	C4A	0.652	7.39E-10	0.940	6.58E-34
3	CTSS	0.595	4.50E-08	0.900	1.41E-26
4	LGALS3BP	0.529	2.12E-06	0.874	2.46E-23
5	APOD	0.561	3.58E-07	0.874	2.54E-23
6	C1QC	0.521	3.18E-06	0.872	3.99E-23
7	**TREM2**	0.567	2.59E-07	0.857	1.37E-21
8	C1QB	0.554	5.54E-07	0.855	2.35E-21
9	MPEG1	0.568	2.33E-07	0.852	4.84E-21
10	**C1QA**	0.548	7.71E-07	0.849	8.53E-21

*Rank, rank of hub gene (ordered descending by CorrGene); gene, gene symbol; CorrAge, Pearson correlation of mouse “yellow” module eigengene with donor age; PvalAve, P-values of correlation in CorrAge; CorrGene, Pearson correlation of mouse “yellow” module eigengene with expression level of the gene in the “Gene” column; PvalGene, P-values of correlation in CorrGene. Genes labeled in bold are microglia signature genes that are shared by the human “black” module and the mouse “yellow” module.*

In conclusion, our analysis identifies co-expressed networks involved in human aging and neurodegeneration that are well preserved in mouse aging and associated with similar molecular pathways. A large part of this conserved regulatory pathway is mediated by microglia. Ultimately, our analyses suggest that the mouse is a valid model system to study changes in microglia during human aging and neurodegeneration.

## Discussion

There is growing evidence suggesting that aging and neurodegenerative diseases show common pathological gene regulation patterns (Chakrabarti and Mohanakumar, 2016). While different cell types are clearly affected differently from one another, the degree to which each cell type is involved in the dysregulation of genetic networks is not entirely understood. Therefore, the overall goal of this study was to identify cell type signature(s) of aging and neurodegenerative diseases by comparing their gene regulatory networks. Through a systematic WGCNA-based analysis, we identified microglia as a major cell type directing gene networks related to aging and three neurodegenerative diseases (AD, HD, and PD) in post-mortem human brains. Moreover, we found that this role of microglia in aging was conserved in mice.

Combining transcriptomic data from different sources and different neurodegenerative diseases enabled us to investigate a large dataset that covered different diseases and a representative age range from 18 to 106 years. Nevertheless, using datasets from different sources often introduces unwanted batch effects. To overcome this hurdle, we leveraged a unique combination of batch-correcting statistical methods (SVA and linear regression models) to segregate the effects of age and neurodegeneration in their respective molecular networks.

We first identified three gene co-expression networks that were significantly correlated to aging and neurodegenerative diseases. Each of these was enriched in genes specific for a distinct cell type, namely neurons (human “turquoise” module), endothelial cells (human “blue” module), and microglia (human “black” module). The association with neurons is consistent with the major feature of neuronal damage, while the relation to endothelial cells could implicate neurovascular dysfunction, which is also seen in aging and neurodegeneration (Kisler et al., 2017). Although a contribution of glial cell types to these conditions was expected, it was surprising to find the strongest association with microglia, while there was only non-significant overlap with astrocytes and no association with oligodendrocytes. Functional annotations of the microglia-associated human and mouse modules showed that it was strongly related to the immune system. Taken together, these results suggest that microglia are the major cell type common to aging and three major neurodegenerative diseases (AD, HD, PD).

Cross-species comparison of gene co-expression networks can provide additional information about network convergence and divergence of networks, which can help to identify key members of the network and species-specific differences (Hawrylycz et al., 2012). As mouse models are widely used in the study of human aging and diseases (Klopstock et al., 2011), we mined mouse aging data to test the cross-species relevance of our finding that microglia signature genes were strongly associated with aging as well as neurodegeneration. In our results, the mouse “yellow” module was significantly preserved between mouse aging and human aging. Furthermore, this module also showed a significant enrichment in microglial genes, suggesting that aging and neurodegenerative disease-associated gene co-expression networks are conserved between mouse and human and associated with microglia.

Comparing the human and mouse microglia-related modules yielded 17 shared genes, of which seven were hub genes: *FCER1G, ITGB2, MYO1F, PTPRC*, and *TYROBP* in human and *C1qa* and *Trem2* in mouse. Recent findings support the role of these genes in aging or neurodegeneration: For example, *C1qa* as part of the complement system mediates early synapse loss in AD, while TYROBP expression is increased in AD and simultaneously increases the expression of AD-related genes such as *Cd33* in an AD mouse model, consistent with its role being a hub gene (Zhang et al., 2013; Herms and Dorostkar, 2016; Haure-Mirande et al., 2018). Furthermore, *TREM2* mutations are well known to increase AD risk in humans, and increased *Trem2* expression in microglia has been found to correlate with amyloid deposition (Brendel et al., 2017). The intersection of hub genes with the Allen Brain Cell Types Database showed that they were indeed strongly expressed in microglia. Consistent with their role as being major gene network drivers, their expression pattern significantly correlated with aging (mouse and human) and neurodegeneration (human). Even though hub genes were present in both modules, they assumed this role in only one of the species and not both. This could indicate a form of network re-wiring (Halfon, 2017), suggesting that the aging- and neurodegeneration-related networks are evolutionarily dynamic. These results are also consistent with previous findings of convergent and divergent pathways in human and mouse brain transcriptomes using a similar approach (Miller et al., 2010).

Several caveats concerning this study such as residual batch effects or tissue heterogeneity should be kept in mind. As is the case for any study that is correlational in nature, inferences that imply a causal relationship must be critically evaluated. Ultimately, biological validation will be required in both mouse and human organoid models of aging, AD, PD, and HD, to ultimately prove their dysfunctional regulation in aging and neurodegeneration.

## Conclusion

In conclusion, we have provided a systems biology-based study that supports two ideas: (i) that there is a common molecular mechanism underlying aging, AD, HD, and PD that might be related to microglia, and (ii) that these microglial gene networks associated with neurodegeneration and aging are conserved in human and mice. Specifically, we have identified seven candidate microglia genes that should be the focus of further investigations, for example in mouse models and human organoid models. Understanding their transition during aging and in neurodegenerative diseases might be of particular interest for future diagnostics.

## Author Contributions

The initial idea for the project was formed by all authors during the 2017 Systems Genetics of Neurodegeneration summer school, Germany. SM performed and guided the majority of data analysis. FS assisted in the analysis and facilitated manuscript submission by preparation of figures. CK created strategies to enable continuation of effective collaboration and prepared main tables for the manuscript. SM, CK, and FS jointly wrote the manuscript. MP-J and JM gave statistical and methodological advice. All authors contributed significantly with regular reviews of the experimental design, interpretation of the results and manuscript preparation.

## Conflict of Interest Statement

The authors declare that the research was conducted in the absence of any commercial or financial relationships that could be construed as a potential conflict of interest.

## Footnotes

1 http://celltypes.brain-map.org/rnaseq
